# X-ray Crystal Structure, Geometric Isomerism, and Antimicrobial Activity of New Copper(II) Carboxylate Complexes with Imidazole Derivatives

**DOI:** 10.3390/molecules23123253

**Published:** 2018-12-09

**Authors:** Ioana Dorina Vlaicu, Gheorghe Borodi, Gina Vasile Scăețeanu, Mariana Carmen Chifiriuc, Luminița Măruțescu, Marcela Popa, Mariana Stefan, Ionel Florinel Mercioniu, Martin Maurer, Constantin G. Daniliuc, Rodica Olar, Mihaela Badea

**Affiliations:** 1 National Institute of Materials Physics, 405A Atomistilor, 077125 Magurele-Bucharest, Romania; ioana.vlaicu@infim.ro (I.D.V.); mstefan@infim.ro (M.S.); imercioniu@infim.ro (I.F.M.); 2 National Institute for Research and Development of Isotopic and Molecular Technologies, 67-103 Donat Avenue, 400293 Cluj-Napoca, Romania; borodi@itim-cj.ro; 3 Department of Soil Sciences, University of Agronomical Sciences and Veterinary Medicine, 59 Mărăşti Str., Sector 1, 011464 Bucharest, Romania; ginavasile2000@yahoo.com; 4 Department of Microbiology, Faculty of Biology, University of Bucharest, 1–3 Aleea Portocalelor Str., 60101 Bucharest, Romania; carmen_balotescu@yahoo.com (M.C.C.); lumi.marutescu@gmail.com (L.M.); bmarcelica@yahoo.com (M.P.); 5 Life, Environment and Earth Sciences Division, Research Institute of the University of Bucharest (ICUB), Spl. Independentei 91–95, 010271 Bucharest, Romania; 6 3S-Pharmacological Consultation & Research GmbH, 1 Koenigsbergerstrasse, 27243 Harpstedt, Germany; dreispharma.harpstedt@t-online.de; 7 Organisch-Chemisches Institut, Westfälische Wilhelms-Universität Münster, Corrensstrasse 40, 48149 Münster, Germany; constantin.daniliuc@uni-muenster.de; 8 Department of Inorganic Chemistry, Faculty of Chemistry, University of Bucharest, 90–92 Panduri Str., 050663 Bucharest, Romania; rodica.olar@chimie.unibuc.ro

**Keywords:** X-ray structure, geometric isomerism, copper(II) complex, imidazole derivative, antimicrobial activity

## Abstract

Five new copper(II) acrylate complexes (acr is the acrylate anion: C_3_H_3_O_2_) with imidazole derivatives (2-methylimidazole/2-MeIm, 5-methylimidazole/5-MeIm, 2-ethylimidazole/2-EtIm) of type: *cis*-[Cu(2-RIm)_2_(acr)_2_]·xH_2_O ((**1**): R = –CH_3_, x = 2; (**4**): R = –CH_2_–CH_3_, x = 0), *trans*-[Cu(2-RIm)_2_(acr)_2_] ((**2**): R = –CH_3_; (**5**): R = –CH_2_–CH_3_) and *trans*-[Cu(5-RIm)_2_(acr)_2_] ((**3**): R = –CH_3_) have been prepared and characterized by elemental analysis, Fourier Transform Infrared spectrometry (FTIR), Electron Paramagnetic Resonance (EPR), electronic reflectance spectroscopy, scanning electron microscopy, and mass spectrometry. The single crystal X-ray diffraction study of complexes (**2**) and (**5**) reveals that the copper(II) ion is located on an inversion center and show elongated octahedral geometry completed by two coplanar bidentate acrylates and two unidentate imidazole derivatives displayed in *trans* positions. For complex (**4**) the single crystal X-ray diffraction shows that the copper(II) ion is in a distorted octahedral environment which can be easily confused with a trigonal prism completed by two bidentate acrylates and two unidentate imidazole derivatives displayed in *cis* positions. These results indicate the fact that complexes (**4**) and (**5**) are the geometric isomers of the same compound bis(acrylate)-bis(2-ethylimidazole)-copper(II). Complexes (**1**) and (**2**), as well as (**4**) and (**5**), were produced simultaneously in the reaction of the corresponding copper(II) acrylate with imidazole derivatives in methanol solution. Furthermore, in order to be able to formulate potential applications of the obtained compounds, our next goal was to investigate the in vitro antimicrobial activity of the synthesized complexes against Gram-positive and Gram-negative bacteria, as well as fungal strains, of both clinical and ecological importance (biodeterioration of historical buildings). The *trans* isomers (**2**) and (**5**), followed by (**4**) have shown the broadest range of antimicrobial activity. In case of (**1**) and (**2**) isomers, the trans isomer (**2**) was significantly more active than cis (**1**), while the cis isomer (**4**) proved to be more active than trans (**5**). Taken together, the biological evaluation results indicate that the trans (**2**) was the most active complex, demonstrating its potential for the development of novel antimicrobial agents, with potential applications in the biomedical and restoration of architectural monuments fields.

## 1. Introduction

Over several decades, copper carboxylate complexes were found to possess many biological functions, such as: antibacterial [1,2], antifungal [1,2,3], cytotoxic and antiviral activities [4], DNA-binding properties [5,6], SOD dismutase mimetic agents [6,7], and antitumor properties [7]. Also, one of the five coordinate, square pyramidal copper-bishistidine complexes was reported to be potentially useful for the treatment of Menkes disease [8]. Top of form bottom of form due to the great versatility of the carboxylate ligand, literature reports a large number of copper(II) complexes with interesting structures, some of them generated by supramolecular interactions [9,10,11]. Furthermore, carboxylate ligands have been often used to generate units which are further used to develop supramolecular architectures [9,12].

Lately, a great interest has gained the synthesis and characterization of complexes with azole type ligands and carboxylates due to their interesting structures and biologic potential, including antimicrobial, antiviral, antidiabetic, and anticancer activities [13].

Copper(II) complex of 2-pyridil-1*H*-benzimidazole has been reported to exhibit a remarkable activity against the growth of different microbial strains, including *Staphylococcus aureus, Staphylococcus epidermidis, Pseudomonas aeruginosa*, *Shigella flexneri*, and *Candida albicans* [14]. Complexes of copper(II) with carboxylate and imidazole ligands have been studied as models for copper proteins that contain both functionalities in the side chain [15,16]. 

The versatility of carboxylate allowed to obtain more than one product from the same synthesis route, in the case of some systems formed from copper(II) acrylate/methacrylate and imidazole. Some of the resulting products were mononuclear bis(acrylate)-bis(imidazole)-copper(II) and bis(methacrylate)-bis(imidazole)-copper(II) with copper(II) ion in a square planar configuration, binuclear tetrakis(μ_2_-acrylate-*O*,*O*′)-bis(imidazole)-dicopper(II) and tetrakis(μ_2_-α-methacrylate-O,O′)-bis(imidazole)-dicopper(II), with a binuclear cage structure and copper(II) ion in a trigonal bipyramidal configuration, and trinuclear copper(II) complexes of the type Cu_3_(CH_2_=CHCO_2_)_5_(OH)(imH)_3_ and Cu_3_[CH_2_=C(Me)CO_2_]_5_(OH)(imH)_3_ with one copper ion in a distorted trigonal bipyramidal configuration and two copper ions in a distorted square-planar configuration [17,18]. 

The α and β forms of bis(imidazole) copper(II) dibenzoate which were obtained by recrystallization from ethanol, although they had different space groups, they belonged the same crystal class and exhibited a similar structure with different geometric parameters, with copper(II) ion in an octahedral stereochemistry with imidazole ligands *trans*-positioned and chelate bidentated benzoates [19]. 

Literature data are scarce concerning geometric isomers *cis* and *trans* for compounds containing both carboxylate and azole type ligands, only one of the two isomers being reported for a certain type of compound like *cis*-bis(1,2-dimethylimidazole)bis(ferrocenecarboxylato)copper(II) and *trans*-bis(ferrocenecarboxylato)-bis(*N*-methylimidazole)copper(II) [20]. 

Our previous studies related to metal carboxylates with azole type ligands [21,22,23] underline the possibility, due to the carboxylate versatility, to obtain from the same synthesis system different compounds. 

This work was carried out as a continuation of our previous research on mixed complexes with acrylate and azole type ligands and we report here a first attempt to obtain both geometric isomers of the same compound in the same synthesis. In this purpose, we synthesized and characterized five new copper(II) acrylate complexes with imidazole derivatives (2-methylimidazole/2-MeIm, 5-methylimidazole/5-MeIm, 2-ethylimidazole/2-EtIm), obtained from three systems: (i) cooper(II) acrylate: 2-methylimidazole: (**1**) [Cu(2-MeIm)_2_(acr)_2_]·2H_2_O blue and (**2**) [Cu(2-MeIm)_2_(acr)_2_] violet; (ii) copper(II) acrylate: 5-methylimidazole: (**3**) [Cu(5-MeIm)_2_(acr)_2_] violet; (iii) copper(II) acrylate: 2-ethylimidazole: (**4**) [Cu(2-EtIm)_2_(acr)_2_] blue and (**5**) [Cu(2-EtIm)_2_(acr)_2_] violet.

An interesting particular feature manifested as geometric isomerism was observed for complexes containing imidazole derivatives substituted in the position 2- of the imidazole nucleus.

As it turned out that the obtained complexes present interesting structural features scarcely reported in literature for such complexes, another objective was to expand the investigations with studies concerning the relationship between isomerism and the biological activity of the obtained complexes, allowing us to formulate potential applications of the obtained compounds. In this regard, our next goal was to investigate the in vitro antimicrobial activity of the synthesized complexes against Gram-positive and Gram-negative bacteria, as well as fungal strains, of both clinical and ecological importance (biodeterioration of historical buildings). 

## 2. Results and Discussion

The interesting chemistry that accompanies metal acrylate complexes with azole type ligands and their promising antimicrobial and anti-biofilm efficiency [21,22,23] focused our interest on them, so in this paper we describe the synthesis, structural characterization, and biological assays related to their possible antimicrobial and anti-biofilm activity.

### 2.1. Synthesis of the Complexes

The first step of the synthesis consisted in copper acrylate obtaining, according to literature data [24]. To a solution of copper(II) acrylate (5 mmol) in methanol or ethanol (20 mL) was added, under continuous stirring, the imidazole derivative (10 mmol) (2-methylimidazole, 5-methylimidazole, or 2-ethylimidazole). After slow evaporation at room temperature of the remaining filtrates, violet (from methanol) and both blue and violet crystals (from ethanol) of compounds (**1**)–(**5**) were isolated.

### 2.2. Characterization of the Complexes

#### 2.2.1. Description of the X-ray Crystal Structures of the Complexes

Complexes crystallize in monoclinic crystal system, (**2**) and (**5**) having *P*2_1_/*c* space group and (**4**) has *C*2/*c* space group. The unit cell parameters for (**2**) and (**5**) are fairly close, while lattice parameters for compound (**4**) are different. Unit cell parameters, other crystal data and structure refinement for complexes (**2**), (**4**), and (**5**) are presented in Table 1. 

**Figure 1:**
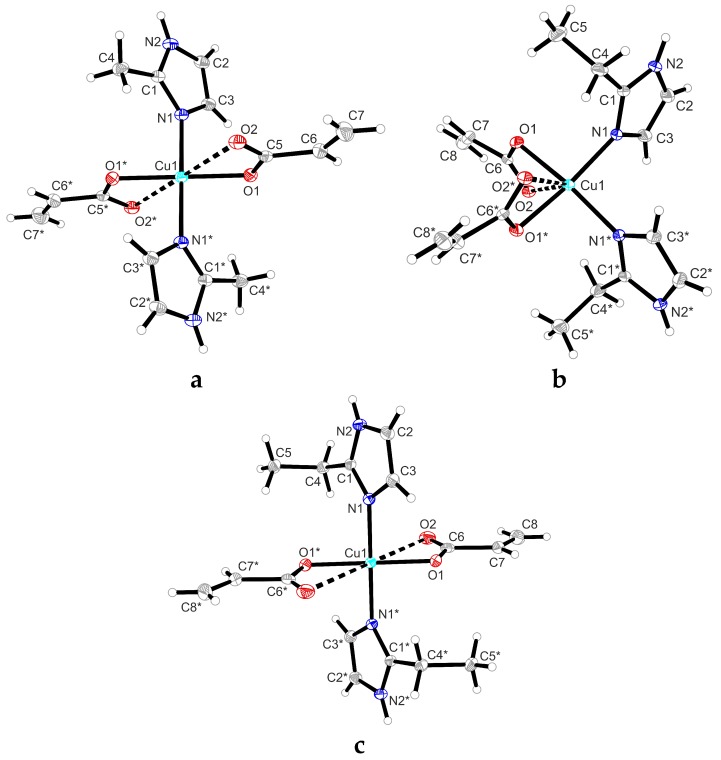
(**a**) Crystal structure of compound (**2**); (**b**) crystal structure of compound (**4**); (**c**) crystal structure of compound (**5**). Thermal ellipsoids are shown at 15% probability. (*) represents the notation of the equivalent atoms in the same type of ligand.

The molecular structure of all complexes consists in two asymmetric units linked each other by inversion operation, the Cu atom being the centre of inversion. The corresponding molecular structures, including the atom numbering are presented in Figure 1. Distances and angles for the three compounds are listed in Table 2.

Complexes (2) and (5) are isostructural (Figure 1b,c) with copper ion adopts a distorted octahedral stereochemistry, being coordinated by two acrylate anions which function as chelate bidentate ligands and two molecules of 2-methylimidazole/2-ethylimidazole as unidentate ligands. The imidazole derivatives are *trans*-positioned. The compounds are monomeric with a centrosymmetric structure, so the CuO_4_ unity in the equatorial plane is perfectly coplanar. The Cu–N bond lengths of 1.986(1)/1.989(1) Å are similar with those reported in literature for this type of compounds [25,26]. For both compounds, the Cu–O bond lengths involving the two oxygen atoms coming from acrylate anion are significantly different [Cu1–O1: 1.969(1)/1.960(1) and Cu1–O2: 2.672(1)/2.857(1) Å]. According to Hathaway criterion [27,28,29] those oxygen atoms in the equatorial planes located at far distance from the central metal ion must be considered as half coordinated. 

**Figure 2:**
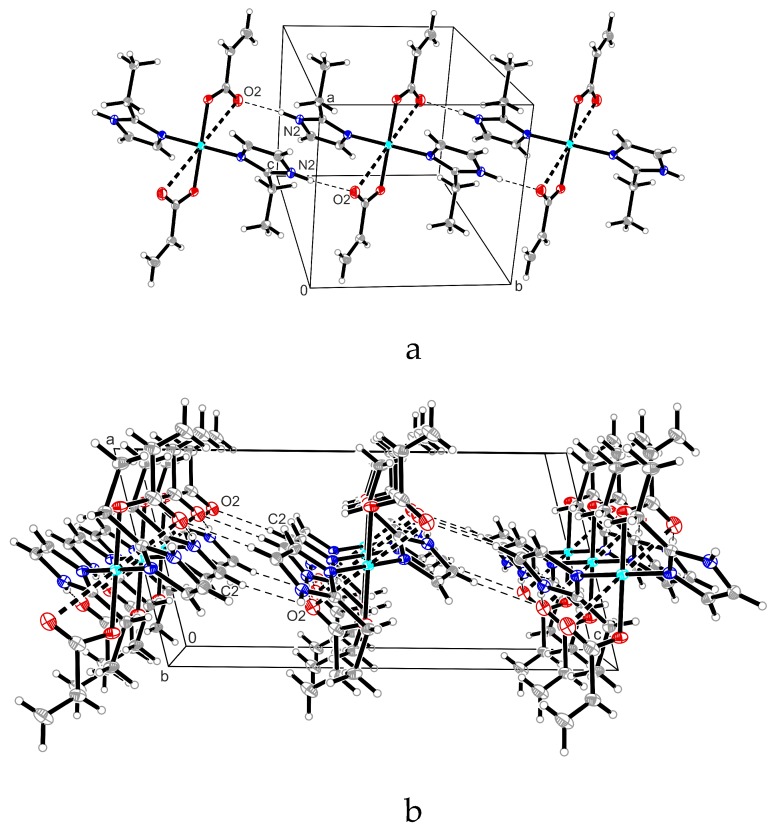
(**a**) Excerpt of the packing diagram of compound (**5**) presenting the linear chain formation along *b*-axis trough double N–H**^…^**O hydrogen bond interactions. (**b**) Excerpt of the packing diagram of compound (**5**) presenting the C–H**^…^**O hydrogen bond interactions between the linear chains along *c*-axis.

Complex (**4**) is monomeric with copper(II) ion in a distorted octahedral stereochemistry and with the chromophore units in a cisoid fashion orientated (see Figure 2a). The two Cu–N bond lenghts [Cu1–N1: 1.985(2)/1.985(2) Å] and two of the Cu–O bonds [Cu1–O1: 1.982(2)/1.982(2) Å] are close to those reported in literature and similar to those evidenced in compound (**5**). The distance between the copper atom and the second oxygen atom from the acrylate unit (Cu1–O2: 2.605(2)/2.605(2) Å) was found slightly shorter compared to the bonds in compounds (**2**) and (**5**). Because of the *cis* arrangement of the ligands, the octahedral stereochemistry is highly distorted. 

As expected, the packing diagram of compounds (**2**) and (**5**) presents similar characteristics. The complexes are stabilized by intermolecular hydrogen bonds between the acrylic O2 atom of one complex moiety and the NH unit of the imidazole derivative of the neighbor complex moiety (N2–H2A**^…^**O2 1.99(2) Å and 171(3)°), forming a linear chain along *b*-axis, as is shown in Figure 2a for complex (**5**). Distances and angles for hydrogen bonds are presented in Table 3.

These linear chains form a 2D network (see Figure 2b) through additional C–H**^…^**O hydrogen bonds between the O2 atom of the acrylate anion and the C–H unit of one another neighbored imidazole ring (C2–H2**^…^**O2 2.609 Å and 133.4°). 

**Figure 3:**
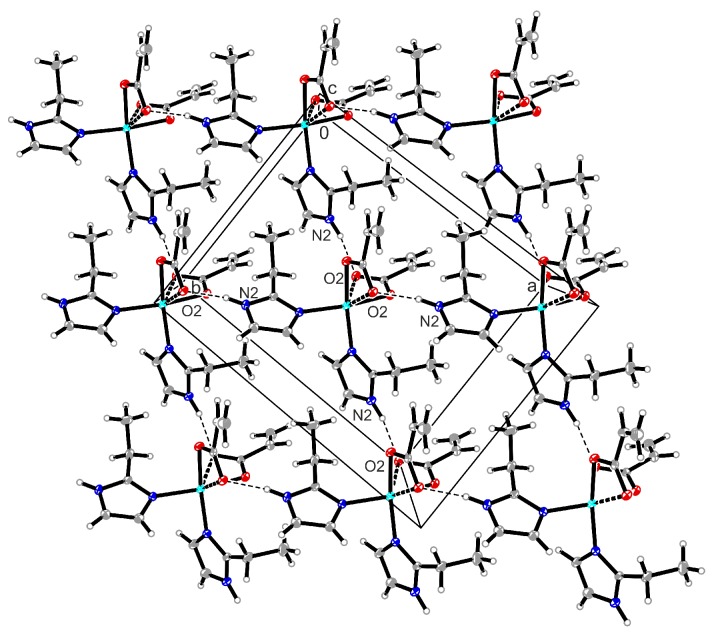
Packing diagram of compound (**4**) presenting the formation of a 2D network. Involving only N–H**^…^**O hydrogen bond interactions.

In contrast, the packing diagram of the cisoid complex (**4**) presents the formation of a 2D network involving only N–H**^…^**O hydrogen bond interactions (N2–H2A**^…^**O2 1.95(2) Å and 166(4)°; see Figure 3). 

#### 2.2.2. Infrared Spectra

The IR spectra for ligands and their copper(II) complexes were recorded (Figure S1) and interpreted; the infrared selected bands for azole ligands (2-MeIm, 5-MeIm, 2-EtIm) and for the complexes (**1**)–(**5**) are listed in Table 4. 

All complexes spectra display the characteristic vibration bands of imidazole type ligands [30]. The fundamental stretching mode characteristic to secondary ammine group ν(NH) appears around 3140 cm^–1^, slightly shifted in the spectra of complexes comparing with the imidazole ligands spectra, indicating that the coordination process occurred and that no deprotonation process took place. In the spectra of complexes, it can be observed an intense band around 1640 cm^–1^, assigned to the vibration mode ν(C=N). This band is shifted toward lower wave numbers with 18–22 cm^–1^ compared to the spectra of imidazole ligands, this indicating that imidazole ligands are coordinated through the N3 nitrogen atom. In the ranges 1530–1580 cm^–1^ and 1330–1240 cm^–1^ it can be observed the characteristic antisymmetric and symmetric stretching modes ν_as_(COO) and ν_s_(COO) of the acrylate anion [31,32]; analyzing the position of these bands and the difference Δ of 175–183 cm^–1^ for all copper complexes, it could be assumed a chelate bidentate coordination mode for acrylate ligands.

The broad band around 3400 cm^–1^ found for complex (**1**) can be assigned to the stretching vibration mode ν(OH) corresponding to the water molecules, their presence being confirmed also by the elemental and thermal analysis. 

These data confirm the presence of imidazole derivatives, bidentately coordinated acrylate as ligands in all five copper complexes and crystallization water molecules in the case of complex (**1**).

#### 2.2.3. UV-Vis-NIR Spectral Data

**Figure 4:**
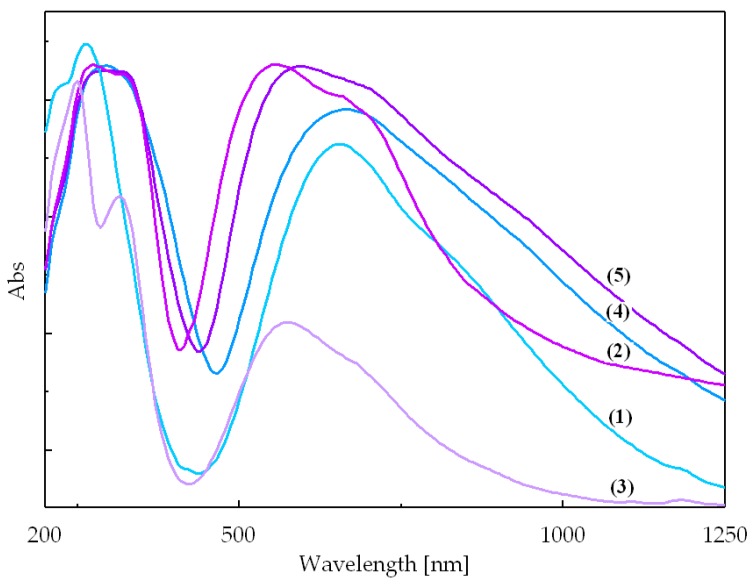
Electronic spectra of complexes (**1**)–(**5**).

Two types of electronic transitions are observed for each of the complexes (Figure 4), meaning intraligand transitions corresponding to the –C=N group in the azole type ligand and d-d transitions corresponding to d^9^ electronic configuration of the divalent copper in an octahedral Jahn-Teller distorted stereochemistry (Table S2) [32]. For all five complexes, the intraligand transitions appear in the range 250–320 nm. A similarity between spectra of complexes (**1**) and (**4**) regarding their intensities, shapes, and maximum absorption positions could be observed. Thus, the broad band centered at about 660 nm corresponds to d_xz,yz_→d_x^2^__−y^2^_ transition for distorted octahedral stereochemistry. Also, it is obvious the similarity between electronic spectra of complexes (**2**), (**3**), and (**5**) with respect to their maximum absorption positions and their shapes. All these compounds present a maximum in the 555–595 nm range and a shoulder at higher wavelengths (675–690 nm) assigned to d_xz,yz_→d_x^2^__−y^2^_ and d_z^2^_→d_x^2^__−y^2^_ transitions. Such a pattern was also observed for other octahedral complexes with *trans*-[CuN_2_O_4_] chromophore [28].

#### 2.2.4. Electron Paramagnetic Resonance Spectral Data

The EPR spectra of the polycrystalline copper(II) complexes (**1**)–(**5**) recorded at 295 K are displayed in Figure S2. The copper complexes (**1**), (**2**), and (**5**) exhibit similar spectra consisting of two resonance lines without any hyperfine splitting, specific for systems with electronic spin S = 1/2, and axial symmetry. The lineshapes are Lorentzian, as expected for compounds where the copper ions are exchange coupled. In the case of complex (3), the parallel component of the axial EPR spectrum displays a resolved hyperfine structure (Figure S2c). The X-band spectrum of complex (**4**) is very broad, with a poor signal-to-noise ratio, while the Q-band spectrum consists of a broad, slightly asymmetrical Lorentzian line, with peak-to-peak linewidth ΔB_pp_ ~ 95.0 mT (Figure S2d). According to Hathaway and Billing [29] such a spectrum could be due to extensive exchange coupling between the four copper(II) ions with misaligned axes present in the unit cell. The small line at ~ 1200 mT (g ~ 2.055) could arise from a very small amount of the isomeric complex (**5**) present in the sample. 

The EPR parameters of all complexes, listed in Table S1, were determined from the lineshape simulation of the EPR spectra, using the spin Hamiltonian for an electron spin S = 1/2 describing the electronic Zeeman interaction of the electron spin S with the external magnetic field B. For complex (**3**), a second term SAI was added, which describes the hyperfine interaction of the electron spin S with the nuclear spin *I* = 3/2 associated to the copper nuclei and A represents the hyperfine coupling constant.

H_S_ = β**SgB** (1)

The small narrow line from the EPR spectrum of complex (**4**) could indeed be fitted with the EPR parameters corresponding to complex (**5**). The g_||_ >> g_⊥_ > 2.04 relationship between the g-values of the complexes (**1**), (**2**), (**3**), and (**5**) indicates a predominantly d_x_^2^_-y_^2^ ground state of the copper(II) ions [27,29], corresponding to an elongated octahedral symmetry, in agreement with the UV–VIS–NIR results. The G = (g_||_—2)/(g_⊥_—2) parameter listed in Table S1 is larger than 4.0, showing that the local tetragonal axes of the copper(II) ions present in the unit cell (Z = 2) in the case of complexes (**2**) and (**5**)) are aligned parallel or only slightly misaligned [28,29]. The best fit of the EPR spectra of these four complexes were obtained for anisotropic linewidths, with Lorentzian lineshape for complexes (**1**), (**2**), and (**5**) and a mixed lineshape, with both Lorentzian and Gaussian contributions, for complex (**3**).

It is also worth noting that, according to [33,34], the g_||_ = 2.322 value points to a more ionic environment of the copper(II) ions in the *cis* complex (**1**), while the g_||_ < 2.3 value of the other three *trans* complexes (**2**), (**3**), and (**5**) with axial spectra would correspond to a covalent character.

#### 2.2.5. Morphology Studies by Scanning Electron Microscopy

**Figure 5:**
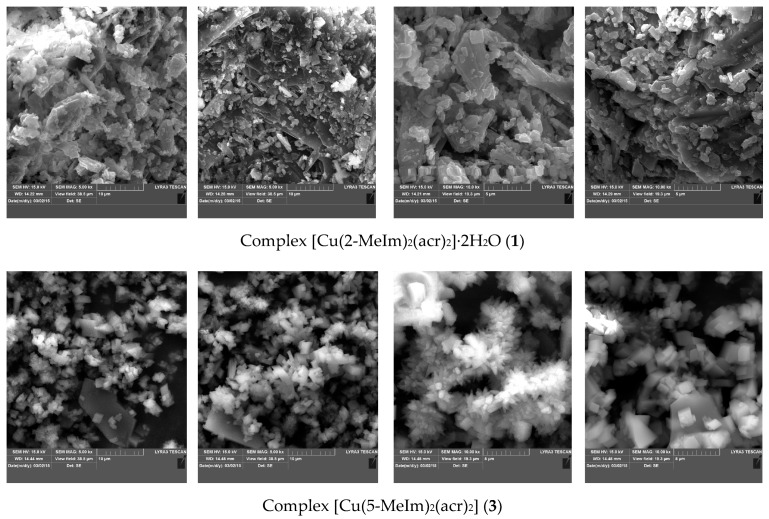
SEM images for complexes (**1**) and (**3**).

For complexes (**1**) and (**3**) the crystallization attempts were not successful for obtaining single crystals suitable for X-ray diffraction analysis. In order to confirm our presumptions about their chemical structure, other techniques being necessary to find more information about their structure. Consequently, Scanning Electron Microscopy (SEM) images were taken revealing that complexes are quite crystalline (Figure 5). For complex (**1**) one can say that it has a very fragile, lamellar, irregular structure. Complex (**3**) crystallites are *nano* single crystals, with a regular cubic structure. 

#### 2.2.6. Mass Spectrometry

Because of the fact that only complexes (**2**), (**4**), and (**5**) could be characterized by single X-ray diffraction analysis, the other two (**1**) and (**3**) were structurally investigated by mass spectrometry, that could provide important information related to a compound composition.

The obtained results were in accordance with the chemical formulations proposed by corelating together the other experimental data provided by chemical and thermal analysis, infrared, electronic, and EPR spectroscopies. 

In the MS + spectra of complexes [Cu(2-MeIm)_2_(acr)_2_] (**1**) and [Cu(5-MeIm)_2_(acr)_2_] (**3**) (Figure S3) it was identified the mass fragment corresponding to the pseudomolecular ion [Cu(MeIm)_2_(acr)_2_ + H]^+^ whose *m/z* is 371 [35]. As it can be observed in the Figure S3, other important peaks that appear in the MS spectra where assigned to the molecular fragments: [Cu(MeIm)_2_(acr)]^+^ (*m/z* = 299), [Cu(MeIm)(acr)_2_ + H]^+^ (*m/z* = 289), [Cu(2-MeIm)(Hacr)]^+^ (*m/z* = 217), and [MeIm + H]^+^ (*m/z* = 83).

#### 2.2.7. Powder X-ray Diffraction

The powder diffraction patterns for all complexes are presented in Supplementary Materials (Figures S4 and S5). For complexes (**2**), (**4**), and (**5**) the powder diffractograms matched well with those simulated from single crystal structure data, indicating that bulk samples were isolated as pure phases. Furthermore, the powder diffractogram for violet complex [Cu(5-MeIm)_2_(acr)_2_] (**3**) displays the same pattern as complexes (**2**) and (**5**) for which a *trans* configuration was revealed from single crystal diffraction.

**Figure 6:**
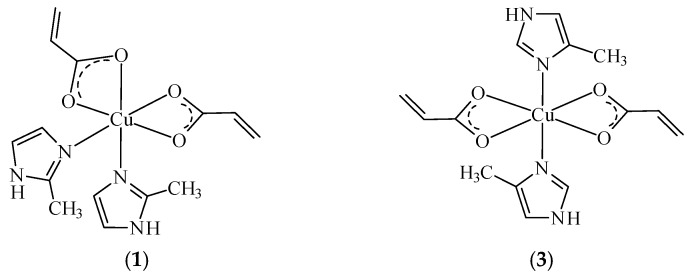
Proposed formulations for complexes (**1**) and (**3**).

Based on all experimental data it was proposed a formulation for complex (**1**) similar to complex (**4**) blue colored with *cis*-octahedral structure and a *trans*-octahedral structure for complex (**3**) similar with (**2**) and (**5**) violet colored (Figure 6).

#### 2.2.8. Antimicrobial Assay

Antibiotic resistance has become a big menace to global health today. Reports are indicating an increase in bacterial resistance, a higher prevalence of opportunistic infections [36,37] reappearance of novel infectious diseases and an acute drop in the availability of active antibiotics. The ESKAPE (***E****. faecium*, ***S****. aureus*, ***K****. pneumonia*, ***A****. baumannii*, ***P****. aeruginosa*, ***E****nterobacter* spp.) group constitutes the most dangerous resistant bacteria, including methicillin-resistant *S. aureus*, extended spectrum beta-lactamase and carbapenemase producing *E. coli,* multidrug-resistant *P. aeruginosa* and vancomycin-resistant enterococci. These bacteria are causing life-threatening infections that cannot be treated with existing antimicrobial agents [36,37], highlighting the acute need to develop new compounds with antimicrobial properties. Moreover, the genetic microbial resistance is amplified by the ability of these bacteria to form biofilms on both human tissues and implanted medical devices; the microroganisms included in biofilms are highly tolerant to host defense mechanisms and to high concentrations of antibiotics [38]. Biodegradation of patrimony objects is a major problem worldwide. Among the biodeteriorating agents, contamination with fungi represents a major problem. Once a biodegradation agent has been isolated and identified, prevention and intervention actions need to be taken. The most frequent interventions are based on the use of different biocidal agents [39]. However, current biocides have a different spectrum of action on microorganisms and often have harmful effects on the patrimony object, for example on stone, causing discoloration, oxidation/reduction of minerals in rock and salt formation, subsequent crystallization by drying, ultimately exfoliating [40]. Therefore, the development of more efficient and safe biocidal agents is required also for the domain of conservation and restauration of cultural heritage.

Several studies reported that the copper(II) complexes exhibit antimicrobial activity, especially against Gram-positive bacteria, which seem to be more susceptible than the Gram-negative ones [41,42,43]. In this study, the antimicrobial activity of the obtained compounds was evaluated against Gram-positive (*Enterococcus faecium, Bacillus subtilis, Staphylococcus aureus*) and Gram-negative (*Escherichia coli, Pseudomonas aeruginosa*) bacteria, as well as against fungi (*Candida albicans, Penicillium* sp. and *Aspergillus* sp.), isolated from clinical samples and from deteriorated historical monuments and compared to that of the ligands. Taking into account that in liquid medium, both isomers are found, we have tested the antimicrobial activity by an adapted diffusion method, consisting in the direct deposition of certain amounts of compounds powders over the layer of microbial culture spread over the solid medium, in order to be able to evaluate and compare the individual antimicrobial activity of each isomer. Also, the antimicrobial activity of the obtained complexes was compared to that of the used ligands.

The copper(II) acrylate exhibited a very good antimicrobial activity against all tested strains, but particularly against two of the most frequently encountered strains in opportunistic infections, including those associated to biofilms, i.e., *S. aureus* and *P. aeruginosa.*

The complexes (**1**) and (**2**) revealed an improved antimicrobial activity, as compared to the used 2-MeIm ligand. Similarly, the complexes (**4**) and (**5**) proved an improved antimicrobial activity as compared to the 2-EtIm ligand against *S. aureus* and *B. subtilis* strains. 

Regarding the comparative antimicrobial activity of the obtained complexes, our results have shown that the *trans* isomers (**2**) and (**5**) exhibited a good antimicrobial activity, demonstrated by production of large growth inhibition zones, followed by isomer (**4**). 

The (**1**) and (**2**) isomers exhibited similar antimicrobial activity against Gram-negative *E. coli* and *P. aeruginosa* strains*,* while in case of the Gram-positive and fungal strains, the trans isomer (**2**) proved to be significantly more active than isomer *cis* (**1**). 

**Figure 7:**
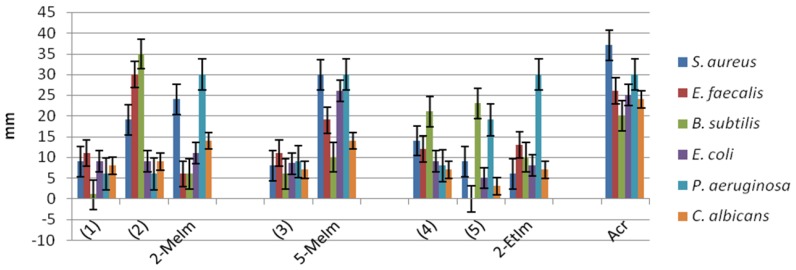
Graphic representation of the growth inhibition zones (mm) produced by the tested complexes (Acr: copper(II) acrylate).

Oppositely, the *cis* isomer (**4**) was demonstrated to be more active than *trans* (**5**), while *P. aeruginosa* and *B. subtilis* strains were inhibited more effectively by the *trans* (**5**) isomer. The activity of *trans* isomer (**3**) was relatively similar against all tested strains, but generally lower than that of the other two trans isomers (Figure 7).

**Figure 8:**
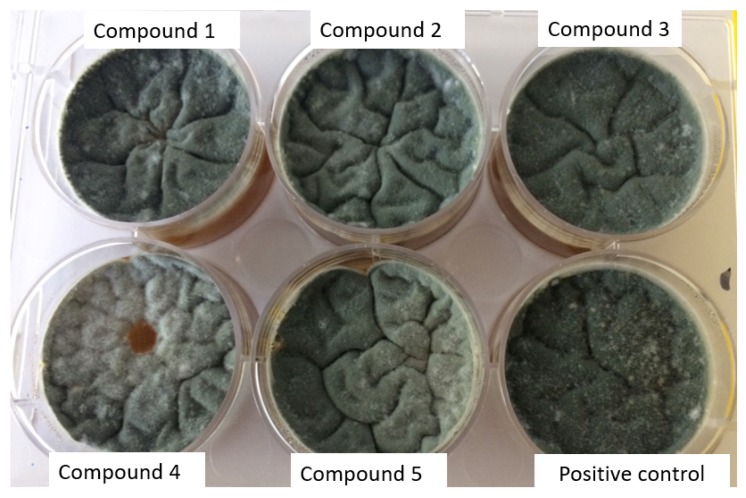
The results of antifungal testing showing the inhibition of mycelium growth (**red disc**) caused by *cis* isomer (**4**). The complexes (**1**), (**2**), (**3**), and (**5**) did not influence the mycelium growth, the fungal growth being similar with positive control.

Fungi have an important role in the discoloration and/or degradation of different architectural monument types, through pigments and organic acids production [44]. *Aspergillus* and *Penicillium* genera have been the most frequently isolated from different historical buildings with different states of degradation in Romania [45]. In this paper, we have also tested the efficiency of the obtained complexes against two strains of these genera, in order to explore their potential for the development of novel and effective biocidal agents for use in the conservation and restauration of tangible cultural heritage. The *cis* isomer (**4**) exhibited a fungicidal effect against the *Penicillium* sp. isolate, as demonstrated by the complete inhibition of mycelium growth (Figure 8). For the same compound, a slight inhibition of sporulation was observed in case of *Aspergillus* sp. isolate. The fungal development inhibition could be due to copper complex binding ability and subsequent inhibition of the enzymatic activity. The compounds (**1**), (**2**), (**3**), and (**5**) did not interfere with sporulation and mycelium growth, the fungal growth being similar with the positive control.

## 3. Experimental Section

### 3.1. General Information

High purity reagents were purchased from Merk Schuchardt OHG (Hohenbrunn, Germany, acrylic acid), Fluka (Saint-Louis, MO, USA, CuCO_3_·Cu(OH)_2_), Sigma-Aldrich (Saint-Louis, MO, USA, imidazole derivatives), were reagent grade, and were used without further purification.

Chemical analysis of carbon, nitrogen and hydrogen has been performed using a PE 2400 analyzer (Perkin Elmer, Waltham, MA, USA). IR spectra were recorded in KBr pellets with a Tensor 37 spectrometer (Bruker, Billerica, MA, USA) in the range 400–4000 cm^−1^. Electronic spectra by diffuse reflectance technique were recorded on solid samples in the range 200–1250 nm, on a V670 spectrophotometer (Jasco, Easton, MD, USA) using Spectralon as standard.

X-ray data for complexes (**2**), (**4**), and (**5**) were collected at room temperature on a SuperNova dual diffractometer with Cu Micro source and EOS CCD detector (Rigaku Oxford Diffraction, Oxford, UK). The crystals were kept at 293K during data collection. Using Olex2 [46], the structure was solved with SHELX [47] structure solution program using Direct Methods and refined with SHELXL [48] refinement package using Least Square minimization. Last refinement step was done using the newer version of SHELXL-2015 [46] as part of the APEX3 software (APEX3 V2016.1-0) [49]. Graphics were done with XP v5.1 [50]. *R*-values are given for observed reflections, and *wR*^2^ values are given for all reflections. For compound (**5**) the acrylate group was found disordered over two positions in the asymmetric unit. Several restraints (SADI, SAME, ISOR, and SIMU) were used in order to improve refinement stability. Moreover, for all three complexes, the hydrogen at N2 nitrogen atom from the imidazole ring was refined freely, but with N-H distance restraints (DFIX).

CCDC-1841956 (**2**), -1841957 (**5**), and -1841958 (**4**) contain the supplementary crystallographic data for this paper. These data can be obtained free of charge from the Cambridge Crystallographic Data Centre via www.ccdc.cam.ac.uk/data_request/cif (3 December 2018).

Powder X-ray diffraction (XRD) patterns were recorded using an XRD-7000 diffractometer (Shimadzu, Kyoto, Japan) with Cu Kα radiation (λ = 1.5406 Å, 40 kV, 40 mA) at a step of 0.2° and a scanning speed of 2 degrees min^−1^ in the 5–60 degrees 2θ range.

Electron paramagnetic resonance (EPR) spectra were recorded at room temperature in the X-band on an EMX Plus spectrometer and in the Q-band on an ELEXSYS E500 spectrometer, both from Bruker, Karlsruhe, Germany. The powder samples were inserted in calibrated pure fused-silica tubes of 2 mm inner diameter. The EPR spectra analysis and simulation were performed with the EasySpin v.5.2.16 program [51].

### 3.2. Synthesis of Complexes

The first step of the synthesis consisted in copper acrylate obtaining, according to literature data [24]: 4 g of basic copper(II) carbonate was mixed with 4.96 mL of acrylic acid for 3 h at room temperature; at the resulted mixture, was added 250 mL methanol and heated under reflux at 50 °C for 2 h. After filtration, the green-blue crystals of copper acrylate were separated from solution by slow evaporation.

To a solution of copper acrylate (5 mmol) in methanol or ethanol (20 mL) was added under continuous stirring the imidazole derivative (10 mmol) (2-methylimidazole, 5-methylimidazole or 2-ethylimidazole). After slow evaporation at room temperature from methanolic solution crystallizes the violet compounds which were filtered off and washed with cold methanol. From ethanolic solution crystallizes on beakers walls blue crystals which were removed and washed with cold ethanol. From the remaining ethanolic filtrates, the violet compounds crystallize further.

From system copper(II) acrylate: 2-methylimidazole (1:1 molar ratio) were obtained two complexes: 

*[Cu(2-MeIm)_2_(acr)_2_]·2H_2_O* (blue polycrystalline solid) (**1**), soluble in dimethylsulfoxide. Anal. Calc.: Cu, 15.66; C, 41.43; H, 5.46; N, 13.80; Found: Cu, 15.45; C, 41.54; H, 5.51; N, 13.93.

*[Cu(2-MeIm)_2_(acr)_2_]* (violet single crystals) (**2**), soluble in alcohols (methanol, ethanol), acetonitrile and dimethylsulfoxide. Anal. Calc.: Cu, 17.18; C, 45.46; H, 4.90; N, 15.15; Found: Cu, 17.25; C, 45.34; H, 4.95; N, 15.33.

From system copper(II) acrylate: 5-methylimidazole (1:1 molar ratio) was obtained complex: 

*[Cu(5-MeIm)_2_(acr)_2_]* (violet polycrystalline solid) (**3**), soluble in alcohols (methanol, ethanol) and DMSO. Anal. Calc.: Cu, 17.18; C, 45.46; H, 4.90; N, 15.15; Found: Cu, 17.21; C, 45.54; H, 5.01; N, 15.29.

From system copper(II)acrylate: 2-ethylimidazole (1:1 molar ratio) after slow evaporation, were isolated two complexes both soluble in alcohols (methanol, ethanol) and dimethylsulfoxide:

*[Cu(2-EtIm)_2_(acr)_2_]* (blue single crystals) (**4**), Anal. Calc.: Cu, 15.97; C, 48.29; H, 5.57; N, 14.08; Found: Cu, 15.91; C, 48.44; H, 5.51; N, 14.15.

*[Cu(2-EtIm)_2_(acr)_2_]* (violet single crystals) (**5**), Anal. Calc.: Cu, 15.97; C, 48.29; H, 5.57; N, 14.08; Found: Cu, 16.11; C, 48.04; H, 5.31; N, 14.15.

### 3.3. Biological Assays

The antimicrobial activity of the complexes was assessed in vitro*,* against five bacterial species: *Bacillus subtilis* ATCC (American Type Culture Collection, Rockville, MD, USA), *Staphylococcus aureus* ATCC 25923, *Escherichia coli* ATCC 25922, *Pseudomonas aeruginosa* ATCC 27853, *Enterococcus faecium* DSM 13590 (Leibniz Institute DSMZ—German Collection of Microorganisms and Cell Cultures) and three fungal strains, i.e., one reference strain of *Candida albicans* ATCC 10231 and two fungal strains isolated from deteriorated cultural heritage monuments—i.e., *Aspergillus* sp., *Penicillium* sp.—using an adapted agar disk diffusion method. The bacteria were routinely cultured on TSA (tryptone soya agar) (Thermo Scientific, Oxoid, UK). From overnight solid cultures, bacterial suspensions with a density of approximately 10^8^ CFU/mL corresponding to McFarland turbidity 0.5 were prepared in sterile physiological solution. After the microbial inoculum was distributed uniformly using sterile cotton swab on a sterile Petri dish Muller Hinton agar, the same amount of the tested complexes powders (10 µg) was distributed on the surface of inoculated agar in a circular spot. The inoculated plates were incubated for 24 h at 37 °C and the antimicrobial activity was read by measuring the growth inhibition zone (expressed in mm) around compound deposition. Filamentous fungal strains represented by two isolates of *Penicillium* sp. and *Aspergillus* sp. were routinely cultivated on PDA (potato dextrose agar) (Sigma-Aldrich). Suspensions of 100.000 spores/ mL were prepared in sterile distillate water. The antifungal testing was carried out in sterile six-well plates. Each well of the six-well plate containing PDA was inoculated with 100 µL spore suspension. The surface of the agar was dried at room temperature before the solid powder of each compound (10 μg) was deposited in the middle, in a circular spot. The inhibition of mycelium growth and sporulation was evaluated after six days of incubation in the dark, at room temperature. All the antimicrobial screening tests were performed in duplicate.

## 4. Conclusions

Five new copper(II) complexes bearing acrylate and imidazole derivatives as ligands have been synthesized and structurally characterized. Their coordination chemistry is very intriguing due to the fact that they possess geometric isomerism. Thus, there was observed this type of isomerism for the couple formed by the compounds *cis*-[Cu(2-MeIm)_2_(acr)_2_]·2H_2_O (**1**) and *trans*-[Cu(2-MeIm)_2_(acr)_2_] (**2**) and the couple formed by the compounds *cis*-[Cu(2-EtIm)_2_(acr)_2_] (**4**) and *trans*-[Cu(2-EtIm)_2_(acr)_2_] (**5**) which crystallized together from the same synthesis and it were possible to isolate from each other.

For the system that contains the ligand 5-methylimidazole, it was possible to obtain only the *trans* conformation. It was observed that the *cis* isomers are blue and the *trans* isomers are violet, so the chromophores are *cis*-[CuN_2_O_4_] (blue) and *trans*-[CuN_2_O_4_] (violet). It is interesting that all new synthesized cooper(II) complexes preferred the octahedral stereochemistry, but taking in account the fact that there is little steric hinderance due to the little volume of the imidazole ligands and the flexibility of the acrylate anion this is very plausible. Until now, in the literature there were *cis*- and *trans*-isomers reported for this type of compound but not for the same system and not for compounds crystallized and isolated from the same synthesis and almost in the same time.

The screening tests revealed that the complex compounds exhibited in vitro antimicrobial activity against bacteria and fungi. The *trans* analog (**2**) and *cis* isomer (**4**) demonstrated that they are the most active compounds, indicating their potential for the development of novel antimicrobial agents with potential applications in the biomedical and restoration of architectural monuments fields.

## Figures and Tables

**Table 1:** Summary of crystal data for complexes (**2**), (**4**), and (**5**).

Compound	[Cu(2-MeIm)_2_(acr)_2_] (2)	[Cu(2-EtIm)_2_(acr)_2_] (4)	[Cu(2-EtIm)_2_(acr)_2_] (5)
Empirical formula	C_14_H_18_CuN_4_O_4_	C_16_H_22_CuN_4_O_4_	C_16_H_22_CuN_4_O_4_
Formula weight	369.86	397.92	397.92
Temperature/K	296(2)	296(2)	296(2)
Crystal system	monoclinic	monoclinic	monoclinic
Space group	*P*2_1_/*c*	*C*2/*c*	*P*2_1_/*c*
a/Å	7.6597(3)	12.9665(7)	7.79135(13)
b/Å	7.6111(2)	9.5520(4)	7.757740(13)
c/Å	15.0825(6)	16.6741(9)	15.6222(3)
α/°	90.00	90.00	90.00
β/°	104.266(4)	114.025(7)	104.2293(18)
γ/°	90.00	90.00	90.00
Volume /Å	852.2(1)	1886.3(2)	915.3(1)
Z	2	4	2
Calculated density /mg/mm^3^	1.441	1.401	1.444
Absorption coefficient /mm^–1^	2.030	1.873	1.930
F(000)	382	828	414
No. of measured and independent reflections	2664/1605	2969/1792	9556/1779
*R*_int_	0.010	0.027	0.029
No. of parameters/restraints	111/1	119/1	148/66
Goodness-of-fit on F2	1.105	1.085	1.056
Final R indexes (*I* ≥ 2σ (I))	R_1_ = 0.029 wR_2_ = 0.085	R_1_ = 0.049 wR_2_ = 0.138	R_1_ = 0.029 wR_2_ = 0.084
Final R indexes (all data)	R_1_ = 0.032 wR_2_ = 0.087	R_1_ = 0.054 wR_2_ = 0.148	R_1_ = 0.031 wR_2_ = 0.086
Largest diff. peak/hole/e Å^−3^	0.23/−0.27	0.48/−0.97	0.19/−0.39
CCDC Nr.	1841956	1841958	1841957

**Table 2:** Selected bond lengths (Å) and angles (°) in (**2**), (**4**), and (**5**).

Bonds	(2)	(4)	(5)
Cu1–O1	1.969(1)	1.982(2)	1.960(1)
Cu1–O1*	1.969(1)	1.982(2)	1.960(1)
Cu1–O2	2.672(1)	2.605(2)	2.857(9)
Cu1–O2*	2.672(1)	2.605(2)	2.857(9)
Cu1–N1	1.986(1)	1.985(2)	1.989(1)
Cu1–N1*	1.986(1)	1.985(2)	1.989(1)
O1–Cu1–N1	91.1(1)	90.3(1)	89.0(1)
O1–Cu1–N1*	89.0(1)	170.9(1)	91.0(1)
O1*–Cu1–N1*	91.1(1)	90.3(1)	89.0(1)
N1–Cu1–N1*	180	93.5(1)	180
O1–Cu1–O1*	180	87.1(1)	180
O2–Cu1–O2*	180	127.9(1)	180
O2–Cu1–N1	90.8(1)	99.8(1)	89.7(3)
O2–Cu1–N1*	89.2(1)	115.5(1)	90.3(3)
O2–Cu1–O1*	125.8(1)	85.9(1)	128.0(2)
O1–Cu1–O2*	125.8(1)	85.9(1)	128.0(2)
O1–Cu1–O2	54.2(1)	55.6(1)	52.0(2)
O1*–Cu1–O2*	54.2(1)	55.6(1)	52.0(2)
O2*–Cu1–N1*	90.8(1)	99.8(1)	89.7(3)
N1–Cu1–O1*	89.0(1)	170.9(1)	91.0(1)
N1–Cu1–O2*	89.2(1)	115.5(1)	90.3(2)
Symmetry operations	(*) 1−*x*, 1−*y*, 1−*z*	(*) 2−*x*, *y*, 1.5−*z*	(*) −*x*, −*y*, 1−*z*

**Table 3:** Distances and angles for hydrogen bonds.

Complex	D–H (Å) N2–H2A	H^…^A (Å) H2A^…^O2	D^…^A (Å) N2^…^O2	<(DHA) (^°^) <(N2–H2A^…^O2)
(**2**) ^(a)^	0.88(2)	1.93(2)	2.784(2)	165(3)
(**4**) ^(b)^	0.87(2)	1.95(2)	2.801(3)	166(4)
(**5**) ^(c)^	0.85(2)	1.99(2)	2.797(1)	171(3)

Symmetry operations: ^(a)^ *x*, *y*−1, *z*; ^(b)^ *x*−1/2, *y*+1/2, *z*; ^(c)^ −*x*, −*y*+1, −*z*+1.

**Table 4:** IR absorption bands (cm^−1^) for ligands and complexes.

2-MeIm	5-MeIm	2-EtIm	(1)	(2)	(3)	(4)	(5)	Assignments
-	-	-	3405 s	-	-	-	-	ν(OH_2_)
3136 m	3136 m	3153 w	3116 s	3125 m	3131 w	3140 m	3134 m	ν(CH), ν(NH)
-	-	-	2944 w	2915 w	2925 w	2920 w	2925 w	ν_as_(CH_2_)
-	-	-	2880 w	2885 w	2888 w	2880 w	2885 w	ν_s_(CH_2_)
1676 m	1676 m	1673 w	1676 w	1630 m	1625 m	1658 w	-	ν(C=N)
1596 vs	1596 vs	-	1613 vs	1605 vs	-	1615 s	1609 vs	δ(NH), ν(CC), ν(CN)
-	-	-	1551 s	1568 vs	1576 vs	1565 vs	1587 vs	ν_as_(COO)
-	-	-	1503 s	1515 m	1520 m	1525 m	1542 m	ν(C=C) aliph
-	-	-	1368 s	1416 m	1387 s	1380 m	1410 vs	ν_s_(COO)
-	-	-	1283 s	1280 w	1290 w	1280 m	1285 m	δ(CH_2_)
1206 w	1206 w	1243 w	1228 s	1215 w	1200 w	1240 w	1245 w	ν(CN), δ(CH)
1155 vs	1155 vs	1153 m	1153 m	1135 w	1112 vs	1158 m	1163 m	ν(CC), ν(CN), δ(CH)
942 s	942 s	956 s	956 m	955 m	963 m	950 w	955 w	δ(CH), δ(imidazole ring)
875 w	875 w	875 w	864 m	870 m	875 m	870 m	879 m	π(CH), δ(imidazole ring)
756 vs	756 vs	751 vs	779 s	760 s	-	755 m	758 m	π(CH)
627 w	627 w	625 w	647 m	650 m	625 m	630 m	624 m	π(NH)

2-MeIm—2-methylimidazole; 5-MeIm—5-methylimidazole; 2-EtIm—2-ethylimidazole; acr—acrylate; (**1**)—[Cu(2-MeIm)_2_(acr)_2_]∙2H_2_O; (**2**)—[Cu(2-MeIm)_2_(acr)_2_]; (**3**)—[Cu(5-MeIm)_2_(acr)_2_]; (**4**)—[Cu(2-EtIm)_2_(acr)_2_]s; (**5**)—[Cu(2-EtIm)_2_(acr)_2_]; ν—stretching; δ—in plane bending; π—out of plane bending; vs—very strong; s—strong; m—medium; w—weak (absorption band intensity).

## Supplementary Materials

The following are available online. Figure S1: IR spectra of complexes (**1**)–(**5**), Figure S2: UV–Vis–NIR spectra of complexes, Figure S3: FAB-MS spectra of complexes (**1**) and (**3**), Figure S4: XRPD patterns for compounds (**2**), (**4**) and (**5**) (in red) shown in comparison with the XRPD pattern of these simulated from SC-XRD data (in black), Table S1: EPR parameters determined for complexes **1**–**5**.
