# Supplementary material for: X-ray Crystal Structure, Geometric Isomerism, and Antimicrobial Activity of New Copper(II) Carboxylate Complexes with Imidazole Derivatives
Source: Molecules. 2018 Dec 9;23(12):3253. doi: 10.3390/molecules23123253 (PMC6321156; doi:10.3390/molecules23123253)
Supplement: Supplementary file 1 [file molecules-23-03253-s001.pdf]

Supplementary materials: X-ray crystal structure and geometric isomerism of new copper (II) carboxylate complexes with imidazole derivatives

Ioana Dorina Vlaicu, Gheorghe Borodi, Gina Vasile Scăețeanu, Mariana Carmen Chifiriuc, Luminița Măruțescu, Marcela Popa, Mariana Stefan, Ionel Florinel Mercioniu, Martin Maurer, Constantin G. Daniliuc, Rodica Olar and Mihaela Badea

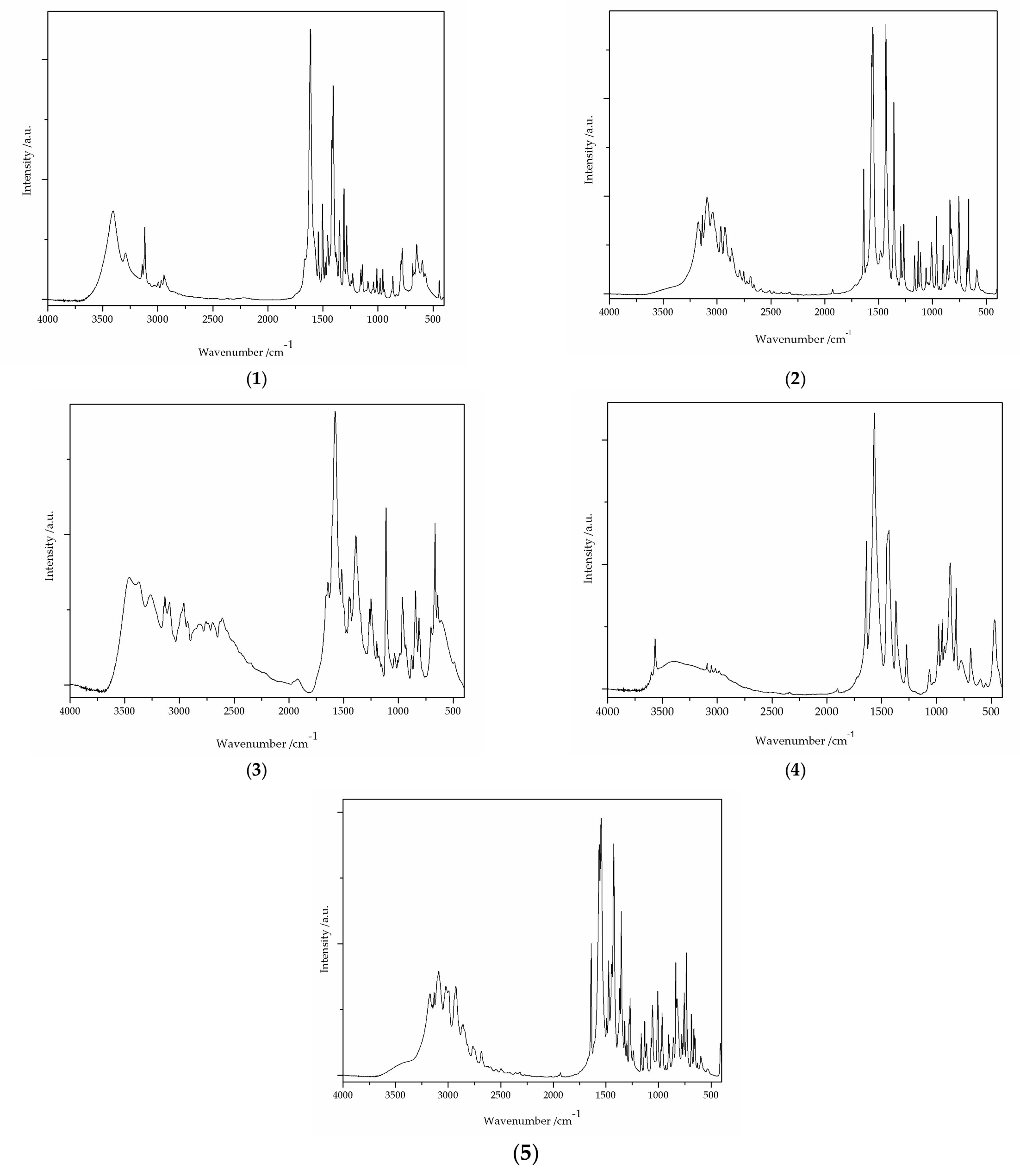

Figure S1. IR spectra of complexes (**1**)-(**5**).

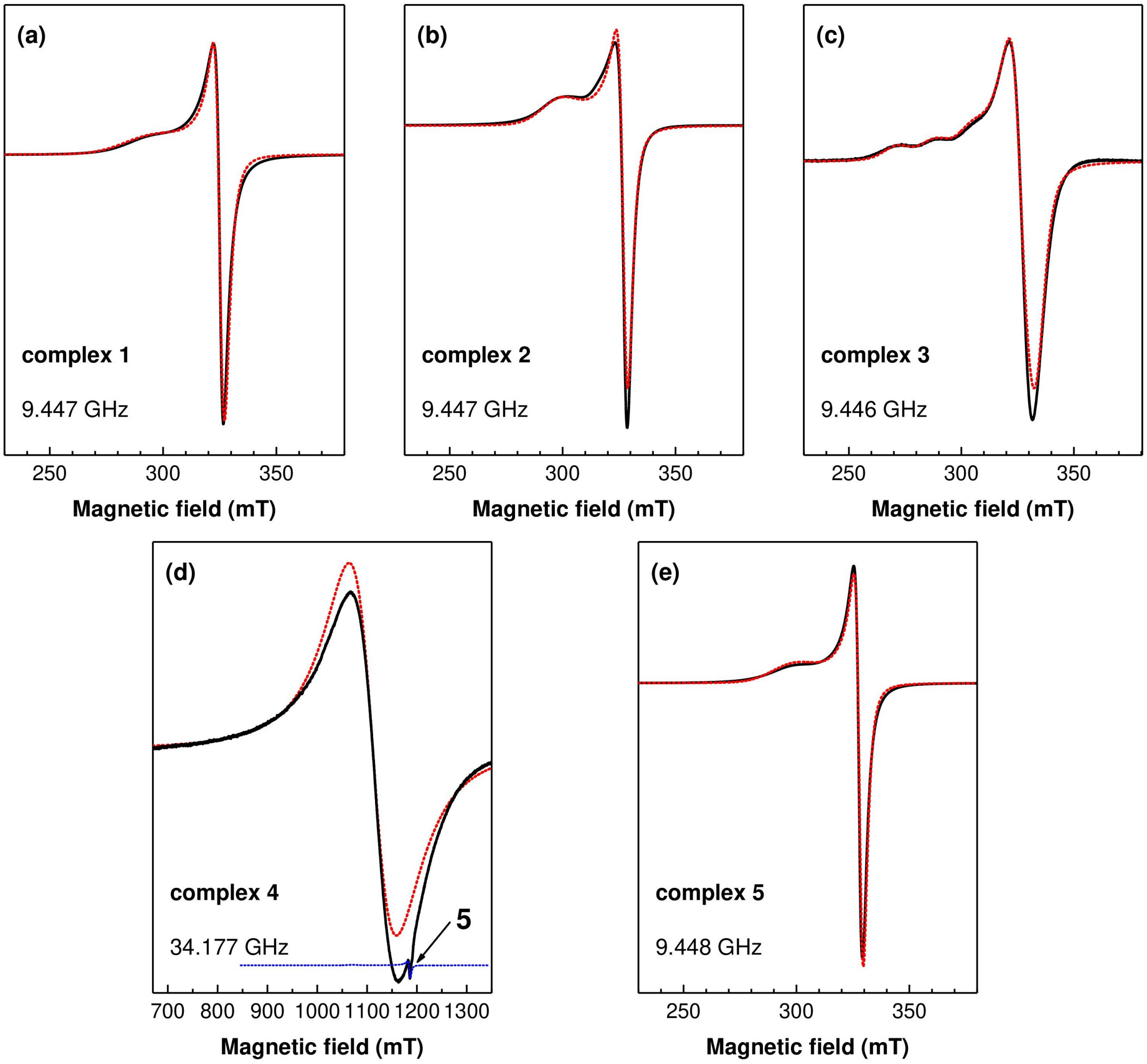

Figure S2. Experimental (solid line) and calculated (dot line) EPR spectra of complexes (**1**)-(**5**).

Table S1. EPR parameters determined for complexes **1**-**5**.

complex	$g_{\parallel}$	$g_{\perp}$	$A_{\parallel}$ [10^{-4} cm^{-1}]	$A_{\perp}$ [10^{-4} cm^{-1}]	G = $(g_{\parallel} - 2)/(g_{\perp} - 2)$
1	2.322 ± 0.003	2.069 ± 0.003	-	-	4.67
2	2.268 ± 0.003	2.057 ± 0.003	-	-	4.70
3	2.277 ± 0.003	2.060 ± 0.003	173 ± 5	14 ± 5	4.62
4	$g_{\parallel} = g_{\perp}$ = 2.197 ± 0.005		-	-	
5	2.273 ± 0.003	2.055 ± 0.003			4.96

Table S2. Absorption maxima in UV-Vis-NIR spectra of complexes (**1**)-(**5**).

**Complex**	**Absorption maxima (nm)**	**Assignments**
[Cu(2-MeIm)$_2$(acr)$_2$]·2H$_2$O **(1)**	260	$\pi \to \pi^*$
	655	$d_{xz,yz} \to d_{x^2-y^2}$
[Cu(2-MeIm)$_2$(acr)$_2$] **(2)**	275<br>320	$\pi \to \pi^*$
	555	$d_{xz,yz} \to d_{x^2-y^2}$
	690	$d_{z^2} \to d_{x^2-y^2}$
[Cu(5-MeIm)$_2$(acr)$_2$] **(3)**	250<br>315	$\pi \to \pi^*$
	575	$d_{xz,yz} \to d_{x^2-y^2}$
	675	$d_{z^2} \to d_{x^2-y^2}$
[Cu(2-EtIm)$_2$(acr)$_2$] **(4)**	295	$\pi \to \pi^*$
	665	$d_{xz,yz} \to d_{x^2-y^2}$
[Cu(2-EtIm)$_2$(acr)$_2$] **(5)**	300	$\pi \to \pi^*$
	595	$d_{xz,yz} \to d_{x^2-y^2}$
	690	$d_{z^2} \to d_{x^2-y^2}$

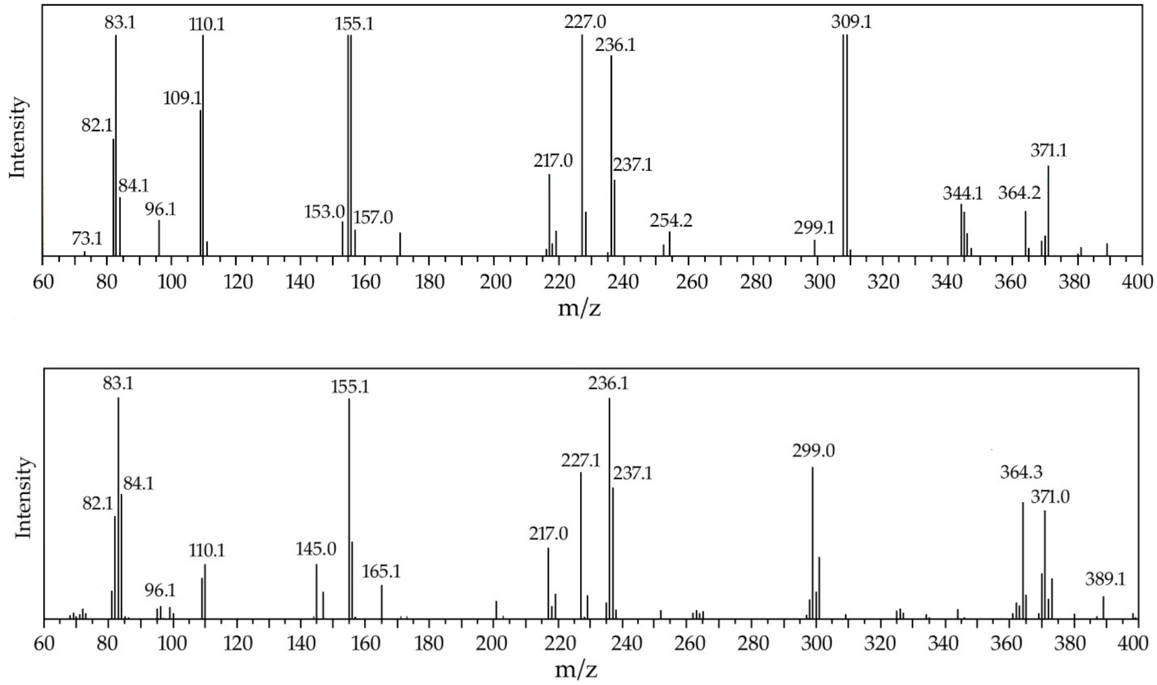

Figure S3. FAB-MS spectra of complexes (**1**) (up) and (**3**) (down).

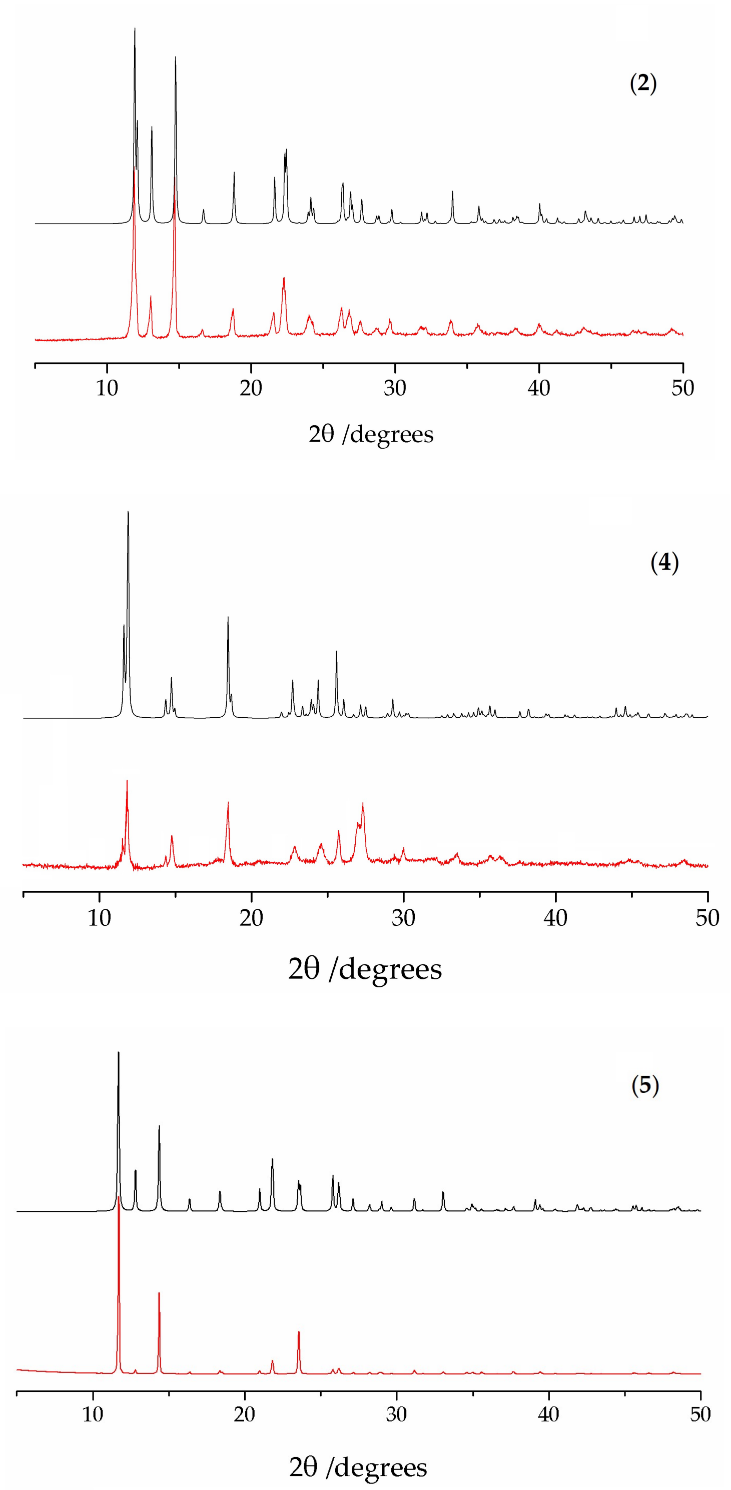

Figure S4. Powder XRD patterns for compounds (**2**), (**4**) and (**5**) (in red) shown in comparison with the simulated XRD from SC-XRD data (in black)

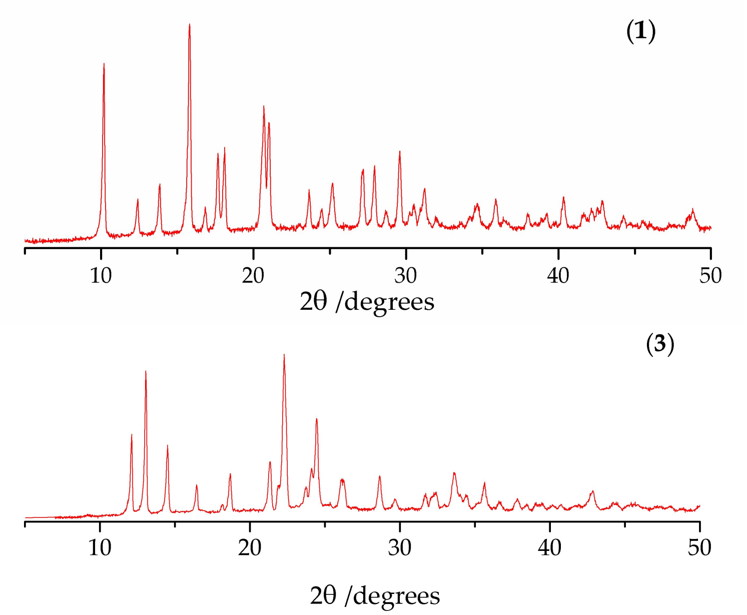

Figure S5. Powder XRD patterns for compounds (**1**) and (**3**)
